# Keratin 6b variant p.Gly499Ser reported in delayed‐onset pachyonychia congenita is a non‐pathogenic polymorphism

**DOI:** 10.1111/1346-8138.14001

**Published:** 2017-08-16

**Authors:** Frances J. D. Smith, W. H. Irwin McLean

**Affiliations:** ^1^ Pachyonychia Congenita Project School of Life Sciences University of Dundee Dundee UK; ^2^ Centre for Dermatology and Genetic Medicine Division of Biological Chemistry and Drug Discovery School of Life Sciences University of Dundee Dundee UK

Dear Editor,

Pachyonychia congenita (PC) is an ultra‐rare hereditary skin disorder primarily characterized by severe, painful and highly debilitating plantar keratoderma, variable hypertrophic nail dystrophy, epidermal cysts, leukokeratosis and other features.^1^ PC is caused by heterozygous dominant‐negative mutations in any one of five keratin genes, *KRT6A*,* KRT6B*,* KRT6C*,* KRT16* or *KRT17* (encoding the differentiation‐specific keratins K6a, K6b, K6c, K16 or K17, respectively).^2^ Due to the rarity of PC, some other genodermatoses with overlapping clinical features can be misdiagnosed as PC.^3^

Our laboratory has run an international molecular genetic diagnostic service for PC in association with the patient advocacy organization Pachyonychia Congenita Project (PC Project; www.pachyonychia.org) since 2004. As of April 2017, we have identified 113 distinct pathogenic *KRT6A*,* KRT6B*,* KRT6C*,* KRT16* or *KRT17* mutations in 774 individuals from 419 unrelated families who are registered in the International Pachyonychia Congenita Research Registry (http://www.pachyonychia.org/pc-data/). With the analysis of a large number of PC patients, the older classification of PC‐1 and PC‐2 subtypes has been shown to be misleading.^1^ Therefore, the nomenclature has recently been revised and a gene‐specific classification is recommended (e.g. PC‐K6a, PC‐K16).^2^, ^3^

Recently, a novel *KRT6B* variant (c.1495G>A; p.Gly499Ser) was reported in *The Journal of Dermatology* as the causative mutation in a patient with late‐onset PC.^4^ We believe that this K6b variant is a non‐pathogenic polymorphism. The p.Gly499Ser variant in *KRT6B* is a common single nucleotide polymorphism (SNP), present in the dbSNP database under the accession number (rs61746355; https://www.ncbi.nlm.nih.gov/projects/SNP/). According to the ExAC database (Exome Aggregation Consortium, Broad Institute; http://exac.broadinstitute.org), in a sample of 60 508 individuals subjected to exome sequencing, 2960 were heterozygous and 57 were homozygous for the minor allele of the SNP rs61746355, p.Gly499Ser. This equates to approximately 5% of the population being either heterozygous or homozygous for the p.Gly499Ser variant in K6b. This high‐frequency SNP is inconsistent with the rarity of PC. Although Guo and colleagues did not observe this SNP in 100 control individuals, who are presumed to be of Chinese ancestry,^4^ it may be that this SNP is very rare in the Chinese population but is common in other ancestral groups.

We would recommend that the authors fully sequence all exons and splice sites of the five keratin genes associated with PC and cross‐reference any detected variations against ExAC or other online SNP databases to identify the actual causative mutation. In addition, given that the phenotype is somewhat unusual and few mutations have been reported in association with delayed‐onset PC previously, it may also be prudent to consider other genes involved with phenotypes that resemble PC. Based on the large number of PC patients in the International PC Research Registry, it is highly unusual for a patient to present only with nail changes, making *GJB6*, and especially *FZD6*, important candidate genes to analyze in this family.^1^, ^5^

## Conflict of Interest

None declared.
